# Avoiding complication of volar locked plates

**DOI:** 10.1186/1753-6561-9-S3-A42

**Published:** 2015-05-19

**Authors:** Marcos Sanmartin Fernandez

**Affiliations:** 1 Department of Orthopaedics and Traumatology, Hospital Povisa, Vigo, 36211, Spain

## 

Volar locking plates are designed to improve and maintain anatomic alignment, even in patients with dorsal comminution and poor metaphyseal bone quality. Its widespread use led to specific complications. Most part is related to tendon, nerve and complex regional pain syndrome [1] and could be avoided by meticulous attention to detail and surgical experience [2].

Tendon and articular injury complications can be prevented by careful avoidance of dorsal cortex and intraarticular screw penetration, use of the low profile plates, and careful placement of the plate proximal to the watershed line.

Most frequent tendons affected are Extensor Policis Longus (EPL) and Flexor Policis Longus (FPL). EPL tendon rupture can occur after a distal radius fractures even when not fixed by a plate [3].

Use of standard intraoperative fluoroscopy imaging is crucial to assess dorsal cortex screw penetration. Lister tubercle can mask prominent screws tips and lateral images have limited sensibility particularly among less experienced observers and for the evaluation of the most ulnar screw positions [4].

Irritation or rupture of flexor tendons, especially the FPL, depends on the position of the plate [5].

Assessment of articular penetration of the screws is critical. Routine use of articular views [6], as well as a 45° pronated oblique view [7] in doubtful cases, is mandatory during placement of each screw.

Postoperative nerve dysfunction is one of the most common complications, especially median nerve dysfunction [1].

K-wires used for provisional and supplementary fixation can be a source of complications. Such complication may be caused by superficial radial nerve irritation due to K-wire placement during fracture reduction.

Finally, loss of reduction due to inadequate screw length or very osteoporotic bone may lead to symptomatic malunion. In extra-articular fractures, a minimum of four locked unicortical distal screws [8] of at least 75% length produce construct stiffness similar to bicortical fixation and should be considered to avoid extensor tendon injury [9]. This is not the case when there are dorsal unstable articular fragments that can displace afterwards.

Once the fracture is healed, plates and screws are not necessary anymore and their removal can be indicated or suggested as a prophylactic measure, as this procedure is usually not associated with complications [10].
